# Temperature-Correlated Changes in Phytoplankton Community Structure Are Restricted to Polar Waters

**DOI:** 10.1371/journal.pone.0135581

**Published:** 2015-08-18

**Authors:** Ben A. Ward

**Affiliations:** 1 Laboratoire des Sciences de l’Environnement Marin, Institut Universitaire Européen de la Mer, Place Nicolas Copernic, Plouzané, France; 2 School of Geographical Sciences, University of Bristol, Bristol, United Kingdom; University of Connecticut, UNITED STATES

## Abstract

Globally distributed observations of size-fractionated chlorophyll *a* and temperature were used to incorporate temperature dependence into an existing semi-empirical model of phytoplankton community size structure. The additional temperature-dependent term significantly increased the model’s ability to both reproduce and predict observations of chlorophyll *a* size-fractionation at temperatures below 2°C. The most notable improvements were in the smallest (picoplankton) size-class, for which overall model fit was more than doubled, and predictive skill was increased by approximately 40%. The model was subsequently applied to generate global maps for three phytoplankton size classes, on the basis of satellite-derived estimates of surface chlorophyll *a* and sea surface temperature. Polar waters were associated with marked decline in the chlorophyll *a* biomass of the smallest cells, relative to lower latitude waters of equivalent total chlorophyll *a*. In the same regions a complementary increase was seen in the chlorophyll *a* biomass of larger size classes. These findings suggest that a warming and stratifying ocean will see a poleward expansion of the habitat range of the smallest phytoplankton, with the possible displacement of some larger groups that currently dominate. There was no evidence of a strong temperature dependence in tropical or sub-tropical regions, suggesting that future direct temperature effects on community structure at lower latitudes may be small.

## Introduction

The structure and function of marine plankton communities are strongly influenced by organism size. Tiny picoplankton (0.2 to 2 *μ*m diameter) comprise a widespread and relatively constant background community that is associated with rapid recycling of nutrients in the microbial loop. The larger nanoplankton (2 to 20 *μ*m) and microplankton (20 to 200 *μ*m) groups are restricted to more productive regions, facilitating both the export of carbon from the atmosphere into the ocean interior [1], and the transfer of energy and biomass to higher trophic levels [2].

The clear positive relationship between ecosystem biomass, productivity and phytoplankton size has been attributed to the strong size-dependence of key plankton traits. In particular, allometric scaling rules suggest that smaller cells benefit from higher resource affinities [3], such that they can exclude larger, less competitive, size classes when nutrients are scarce [4]. Larger groups can only avoid competitive exclusion where nutrients accumulate, either as a consequence of an intermittent nutrient supply [5], or because top-down control by zooplankton and viruses prevents full nutrient drawdown by the smaller groups [6, 7].

This view has been implicitly incorporated into diagnostic models of phytoplankton community structure, through which the relative chlorophyll *a* biomasses of pico-, nano- and microplankton are estimated as a function of total community chlorophyll *a* [8–10]. Larger size classes are assumed to become established alongside smaller ones, and hence picoplankton are typically shown with relatively unrestricted biogeography from low to high latitudes. It is, however, important to note that while semi-empirical models based on total chlorophyll *a* concentrations [8, 9] typically assume that phytoplankton communities are organised solely as a function of total productivity and biomass, community structure has also been shown to vary as a function of temperature [11], and clear exceptions to general chlorophyll-based trends have been recorded at high latitudes [8]. Measurements along a meridional transect in the Ross Sea, for example, have shown a counterintutive shift towards larger cells with increasing nutrient stress and decreasing temperatures polewards of 60°S [12]. More recently, warmer temperatures in the Arctic have been associated with increased abundance of picoplankton at the expense of nanoplankton, while total chlorophyll concentrations remained unchanged [13]. Similarly, measurements in the northwest Atlantic have shown a temperature-dependent increase in the mean size of the picoplankton that appears to be driven by the exclusion of very small cells in colder waters [14]. At the global scale, a reanalysis of ∼70,000 *Prochlorococcus* and *Synechococcus* abundance measurements showed a strong decline in the abundance of these two picoplankton groups at cold temperatures and high latitudes, but found only a weak relationship with nutrient availability [15].

In this study, a global dataset of size-fractionated chlorophyll *a* and temperature measurements [4] is used to show that an existing, temperature-independent, model of phytoplankton community size structure [9, 16] tends to overestimate picoplankton chlorophyll *a* concentrations at temperatures below ∼2°C. From this standpoint, temperature dependence is incorporated into the model, allowing a significant improvement in the model’s ability to represent and predict cold-water chlorophyll *a* concentrations in all three phytoplankton size classes. The observed trends are subsequently extrapolated to the global scale using satellite estimates of sea surface temperature and chlorophyll *a* concentration, and the ecological and biogeochemical implications are discussed.

## Observations and models

**Fig 1:**
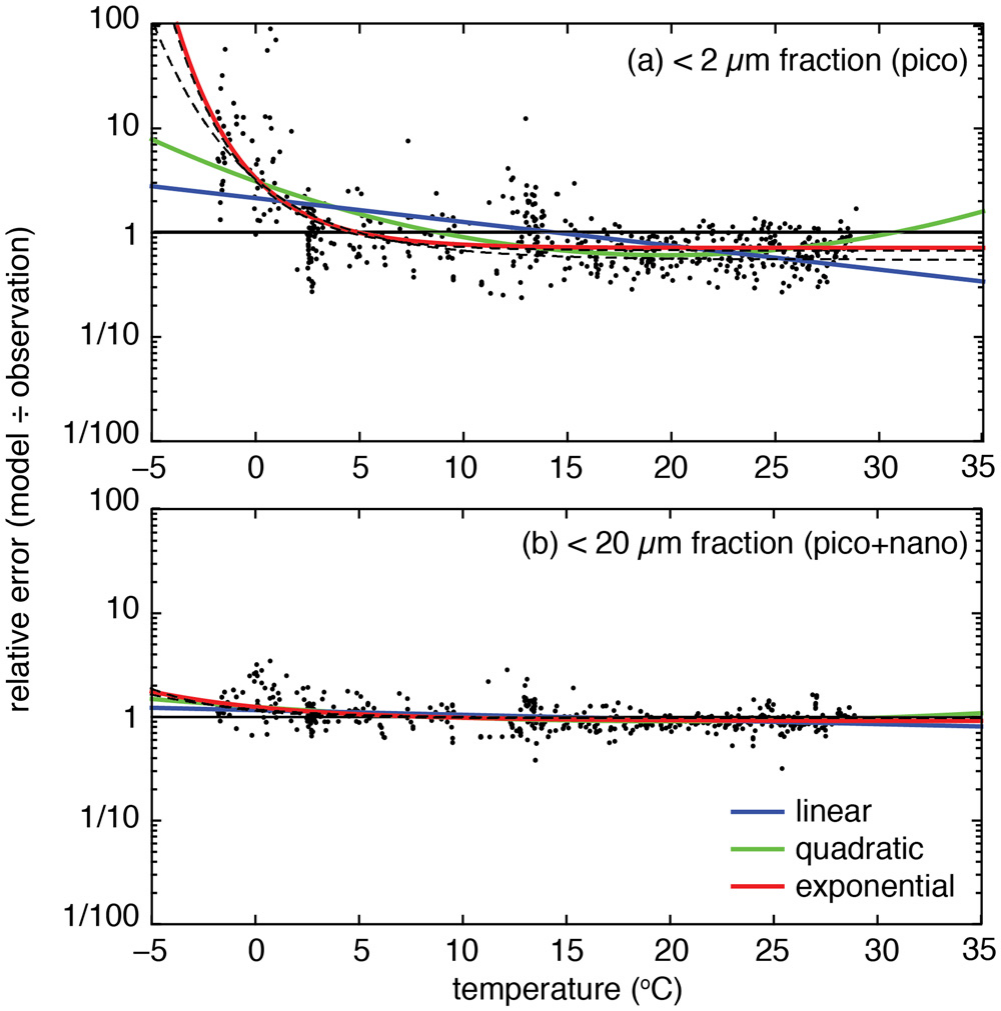
Relative errors of the temperature-independent model, plotted as a function of temperature. The three coloured lines in each plot represent linear (blue), quadratic (green) and exponential (red) functions that were fit to the residuals as a function of temperature (Table 2). The dashed black lines indicate the fit of the exponential function, with the exclusion of all observations taken below either 15 or 40 m depth. Data were generously made available by E. Marañón [4].

A total of 620 concurrent depth-resolved observations (Table 1) for water temperature and size-fractionated chlorophyll *a* biomass (picophytoplankton, nanophytoplankton and microphytoplankton) were taken from a previously published database [4] (see also [11, 17]). The observations span latitudes from 52°N to 68°S, across a temperature range of more than 30°C (-1.8°C to 28.9°C). Contrary to these earlier studies, the dataset used here includes all available measurements regardless of depth (maximum depth was 170 m), because it was found that limiting the observations to those taken above either 40 or 15 m had very little effect on the results (see Fig 1 and legend). Seven observations from the highly eutrophic and coastal Straits of Johor and Singapore Strait were excluded on the grounds that they are clear outliers from the rest of the data (the seven points located above and to the right of the main sequence in Fig 1A of reference [11]).

**Table 1:** Index of the studies from which the size-fractionated chlorophyll *a* and temperature measurements were taken. All data were compiled by [4].

Project	Subset	$\bar{T}\pm \sigma$	*n*	Source
Rothera Time Series	Winter	-1.7 ± 0.08	7	[18]
Rothera Time Series	Autumn	-1.3 ± 0.38	13	[18]
Rothera Time Series	Spring	-1.1 ± 0.66	26	[18]
SSAAC	-	0.0 ± 0.13	6	[19]
SAAMES	Marginal ice zone	0.2 ± 0.15	4	[20]
Rothera Time Series	Summer	0.8 ± 0.36	12	[18]
Southern Ocean	-	1.4 ± 1.01	10	[21]
SOIREE	Inside patch	2.6 ± 0.13	36	[22]
SOIREE	Outside patch	2.6 ± 0.10	14	[22]
Station KNOT	Winter	2.7 ± 0.99	2	[23]
SAAMES	Polar Front	4.4 ± 2.84	8	[20]
SEEDS	Inside patch	6.3 ± 2.66	28	[24]
SEEDS	Outside patch	6.7 ± 2.57	19	[24]
AMT 2	Temperate	10.5 ± 2.58	19	[25]
AMT 3	Temperate	11.1 ± 5.26	32	[25]
Station KNOT	Summer	11.1 ± 4.09	4	[23]
Station KNOT	Autumn	11.6 ± 0.42	2	[23]
TPR project	-	14.3 ± 1.66	94	[26]
AMT 3	Oligotrophic	21.2 ± 3.13	81	[25]
AMT 2	Oligotrophic	21.7 ± 3.29	59	[25]
AMT 2	Equatorial	23.1 ± 5.27	29	[25]
AMT 3	Equatorial	24.2 ± 4.09	40	[25]
Tehuanos II	-	24.8 ± 1.49	7	[27]
Arabian Sea	-	25.9 ± 2.26	68	[28]

Abbreviations: SSAAC—Scandinavia-South Africa Antarctic Cruise; SAAMES—South African Antarctic Marine Ecosystems Study; SOIREE—Southern Ocean Iron RElease Experiment; KNOT—Kyodo North Pacific Ocean Time-series; SEEDS—Subarctic-Pacific iron Experiment for Ecosystem Dynamics Study; AMT—Atlantic Meridional Transect; TPR—Tamaño, Producción y Respiración del Fitoplancton.

### Existing temperature-independent model

Following previous authors [16, 29], Brewin *et al.* [9] developed a semi-empirical model through which the log-transformed chlorophyll *a* biomass in the picoplankton and combined picoplankton+nanoplankton size classes (subscript *s* = pico or nano,pico) can be estimated solely as functions of *C*_total_.

$$\log_{10}\left(C_{s}\right)=\log_{10}\left(C_{s}^{m}\right)+\log_{10}\left(1-e^{-\frac{D_{s}}{C_{s}^{m}}C_{\text{total}}}\right)$$ (1)

Note that while Eq 1 is functionally identical to Eqs 13 and 15 in reference [9], the inverse of *C*_*s*_ is here included in the exponent [29, 30]. This allows *D*_*s*_ to constrain the relative contribution of *C*_*s*_ to *C*_*total*_ at low total chlorophyll concentrations (*C*_*total*_ → 0), while $C_{s}^{m}$ independently describes the maximum value of *C*_*s*_ at high total chlorophyll concentrations (*C*_*total*_ → ∞). The log transformation was applied to reflect the fact that chlorophyll *a* concentrations in the ocean follow an approximately log-normal distribution, and to avoid excessive influence of large chlorophyll *a* concentrations on the model parameters. These were estimated from the data by linear least-squares regression, using the “fit” package in Matlab (R2012a). Prior parameter constraints were applied in accordance with the ecological meaning of the parameters ($C_{s}^{m}\geq 0$ and 0 ≤ *D*_*s*_ ≤ 1). Chlorophyll *a* biomass within the nano and micro size fractions were estimated under the assumption that the total biomass is equal to the combined biomass of all three size fractions (*C*_micro_ = *C*_total_ − *C*_nano,pico_ and *C*_nano_ = *C*_nano,pico_ − *C*_pico_).

### Temperature-dependence

For each of the two directly estimated size fractions (*s* = pico and nano,pico), the relative residual errors (*ϵ*_*s*_ = model ÷ observation) of the calibrated model are plotted as a function of temperature in Fig 1. While the model residuals show no temperature-dependence in waters warmer than approximately 2°C, there is an increasing tendency to overestimate picoplankton chlorophyll as temperatures fall below this threshold (Fig 1a). A similar but much weaker trend is apparent at cold temperatures in the combined picoplankton and nanoplankton size class (Fig 1b).

The trends in Fig 1 suggest that the addition of a temperature-dependent term would allow the size class model to better reproduce the observations at low temperatures. The appropriate form for this additional term was examined by fitting three alternative (linear, quadratic and exponential) temperature-dependent functions to the log-transformed model residuals, as outlined in Table 2.

**Table 2:** Coefficients and goodness-of-fit statistics for the functions fit to the temperature-independent model residuals (*ϵ*_*s*_), as shown in Fig 1. Goodness-of-fit metrics: SSE = Sum of Squared Errors; r^2^ = coefficient of determination (significant at p = 0.01); RMSE = Root Mean Squared Error.

Function	*a*_*s*_	*b*_*s*_	*c*_*s*_	SSE	*r* ^2^	RMSE
log_10_(*ϵ*_pico_) =						
*b*_*s*_ *T* + *c*_*s*_	-	-0.023	0.33	83.00	0.26	0.37
*a*_*s*_ *T* ^2^ + *b*_*s*_ *T* + *c*_*s*_	0.0018	-0.072	0.49	71.24	0.37	0.34
*a*_*s*_ *e* ^−*b*_*s*_*T*^ + *c*_*s*_	0.67	0.31	-0.15	62.12	0.45	0.32
log_10_(*ϵ*_nano,pico_) =						
*b*_*s*_ *T* + *c*_*s*_	-	-0.0045	0.065	7.96	0.13	0.11
*a*_*s*_ *T* ^2^ + *b*_*s*_ *T* + *c*_*s*_	0.00035	-0.014	0.096	7.54	0.17	0.11
*a*_*s*_ *e* ^−*b*_*s*_*T*^ + *c*_*s*_	0.14	0.15	-0.039	7.57	0.17	0.11

The resulting fits are shown in Fig 1, with coefficients and goodness-of-fit metrics given in Table 2. While the linear function has a weak but significant negative slope for both size fractions (95% confidence level), it is outperformed in both cases by the quadratic and exponential functions. Between these two higher order functions, there is little to differentiate them with regard to the picoplankton + nanoplankton residuals, but the exponential function is clearly the best in terms of reproducing the picoplankton residuals. Given its good overall performance, and the fact that it better captures lack of a clear trend in warm waters, the exponential form was used to incorporate temperature dependence into Eq 1.

$$\log_{10}\left(C_{s}\right)=\log_{10}\left(C_{s}^{m}\right)+\log_{10}\left(1-e^{-\frac{D_{s}}{C_{s}^{m}}C_{\text{total}}}\right)-a_{s}e^{-b_{s}T}$$ (2)

The model parameters were estimated as for the temperature-independent model. An additional constraint (*a*_*s*_ ≥ 0) ensured that the estimated biomass in each size fraction did not exceed $C_{s}^{m}$.

## Results

**Fig 2:**
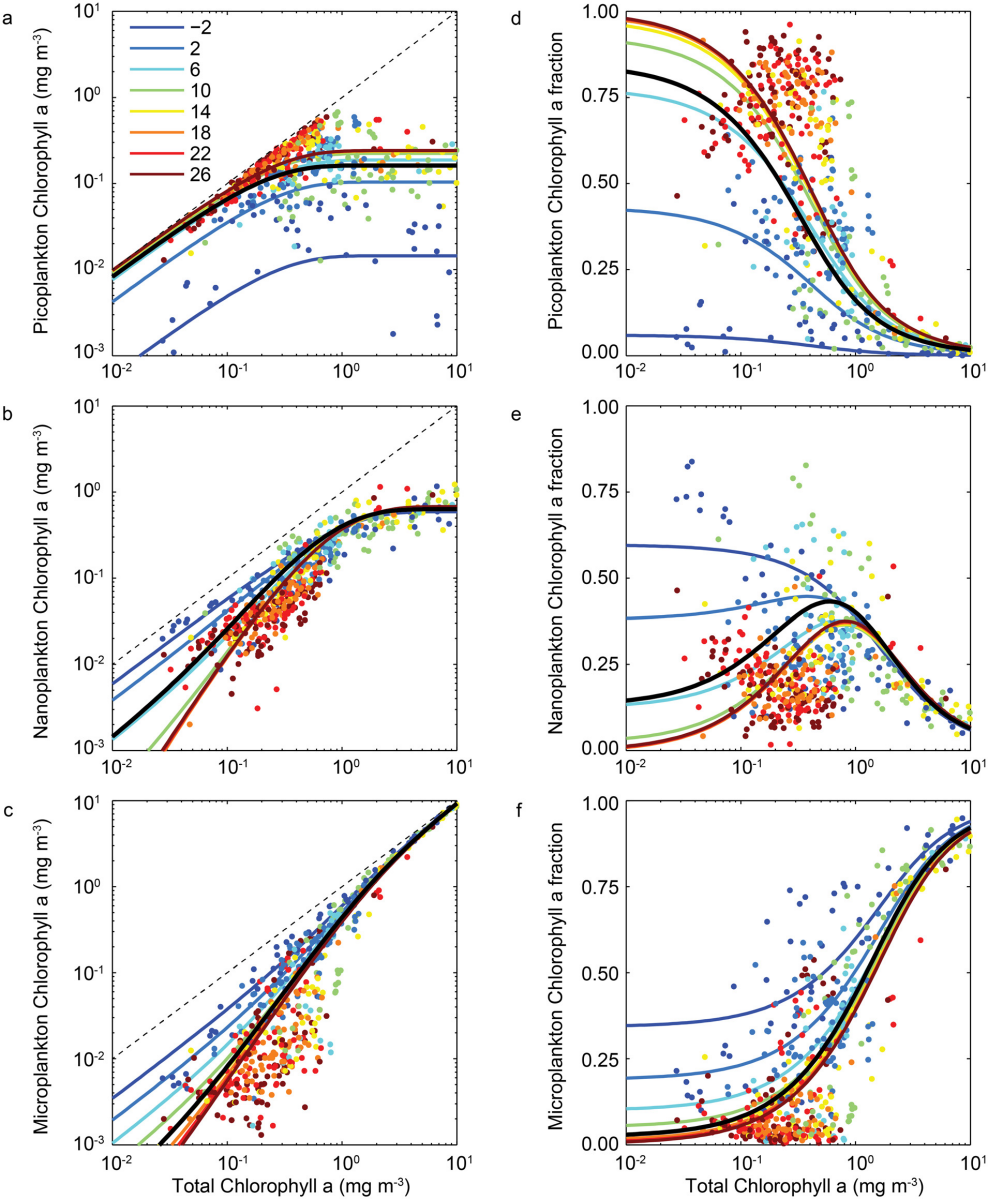
Absolute (left) and relative (right) chlorophyll *a* biomass in (a,d) picoplankton, (b,e) nanoplankton and (c,f) microplankton size-classes, as a function of total chlorophyll *a* (*x*-axis) and temperature (colours). The thick black lines represent the best-fit temperature-independent functions (Eq 1). The coloured lines represent the best-fit temperature-dependent functions (Eq 2) at discrete temperatures (see legend).

The observations and resultant calibrated functions for the pico-, nano- and micro-plankton size classes are plotted as a function of total chlorophyll *a* and temperature in Fig 2a–2c. The same observations and functions are plotted in terms of the fractional contribution of each size class to total biomass in Fig 2d–2f. Coefficients and goodness-of-fit metrics for the temperature-independent and temperature-dependent models are shown for each size fraction in Table 3.

**Table 3:** Coefficients and goodness-of-fit statistics for the temperature-independent (Eq 1) and temperature-dependent (Eq 2) functions. pSSE = predictive SSE from cross-validation experiment; AIC = Akaike Information Criterion. Coefficients of determination are significant at p = 0.01.

Function	$C_{s}^{m}$	*D*_*s*_	*a*_*s*_	*b*_*s*_	SSE	*r* ^2^	RMSE	pSSE	AIC
log_10_(*C*_pico_) = f(*C*_total_)	0.162	0.849	-	-	112.5	0.21	0.43	121.8	11254
log_10_(*C*_pico_) = f(*C*_total_, *T*)	0.2414	1.00	0.668	0.302	62.1	0.56	0.32	74.8	6218
log_10_(*C*_nano,pico_) = f(*C*_total_)	0.794	0.976	-	-	9.11	0.88	0.12	9.43	9114
log_10_(*C*_nano,pico_) = f(*C*_total_, *T*)	0.921	1.00	0.129	0.173	7.57	0.90	0.11	8.62	7578
log_10_(*C*_nano_) = f(*C*_total_)	-	-	-	-	57.3	0.78	0.30	63.6	-
log_10_(*C*_nano_) = f(*C*_total_, *T*)	-	-	-	-	44.2	0.80	0.27	46.5	-
log_10_(*C*_micro_) = f(*C*_total_)	-	-	-	-	136.5	0.83	0.47	143.8	-
log_10_(*C*_micro_) = f(*C*_total_, *T*)	-	-	-	-	114.3	0.86	0.43	116.1	-

### Temperature-independent relationships

The temperature-independent model (Eq 1) conforms to the relationships between size-fractionated chlorophyll *a* and total chlorophyll *a* found by [9]. The picoplankton function accounts for a large fraction (*D*_pico_ = 0.85) of low total chlorophyll *a* concentrations, but saturates at a maximum value of $C_{\text{pico}}^{m}=0.162$ mg chlorophyll *a* m^−3^, and thus represents a very small fraction of the total community at high total biomasses. The monotonic function cannot reproduce the decline in picoplankton biomass that is seen in the data at very high total biomasses (although the declining trend may be somewhat artificial, as it is not seen if picoplankton biomass is measured in terms of carbon [31]).

The nanoplankton function initially represents a much smaller fraction (0.13) of the total community biomass, but rapidly increases in both absolute and relative terms, to briefly dominate the community at intermediate biomasses. The nanoplankton function eventually saturates at a maximum value of just over 0.6 mg chlorophyll *a* m^−3^, such that it also represents a small fraction of high total biomasses.

The microplankton are extremely scarce at low total biomass (∼2.4%), but increase very rapidly across the entire range of total biomass, eventually reaching concentrations of ∼10 mg chlorophyll *a* m^−3^ within the observed range. This increasing trend leads to the total dominance of microplankton in high biomass systems, although this may also be, at least in part, an artefact of filter clogging, with some nanoplankton incorrectly retained in the microplankton fraction [31].

The temperature-independent model explains just over 20% of the variance of picoplankton biomass, rising to 78 and 83% for the nanoplankton and microplankton, respectively (Table 3).

### Temperature-dependent relationships

At any given temperature, estimates of size-fractionated chlorophyll *a* from the temperature-dependent model (Eq 2) are qualitatively similar to those of the temperature-independent model. There are however clear temperature effects, especially at cold temperatures, and these are particularly clear when viewed in terms of the biomass fractions (Fig 2d–2f). The picoplankton function varies relatively little between 10 and 30°C, but there is a marked reduction in picoplankton chlorophyll *a* at lower temperatures. For any given total chlorophyll *a* concentration, picoplankton biomass may be up to an order of magnitude lower in sub-zero waters, relative to tropical latitudes. In very low biomass systems the picoplankton community fraction declines by almost 100% from warm to very cold waters.

In contrast, for any given total biomass, cold waters support a larger proportion of both nanoplankton and microplankton, especially in low to intermediate biomass systems. Below 1 mg total chlorophyll *a* m^−3^, the biomass within either size class may be between 3 and 10 times higher at 0°C relative to 10 or 30°C. Among highly oligotrophic systems, the nanoplankton and microplankton size-fractions are very scarce in warm waters, but together completely dominate communities where the temperature is less than 5°C.

Adding temperature dependence explains a further 35% of the variance in picoplankton chlorophyll *a*, relative to the temperature-independent function (21 to 56%). The same statistic is improved by only 2% for the nanoplankton (78 to 80%), and by 3% for the microplankton (83 to 86%). In terms of the (interdependent) fractional contributions to total chlorophyll *a*, the temperature dependent function explains an additional 24% of variance in the picoplankton fraction (42 to 66%) and a further 18% for the nanoplankton fraction (8 to 26%). The statistic is improved by only 6% for the microplankton fraction (74 to 80%).

The parameter *D*_*s*_ describes the contribution of *C*_*s*_ to *C*_*total*_ at low total chlorophyll concentrations, and takes the upper bound value of 1 in the temperature-dependent model for both *C*_pico_ and *C*_pico+nano_. This indicates two-things. Firstly, in warmer waters where the temperature effect is negligible (effectively *T* > 5°C), picoplankton biomass tends towards the total community biomass as total biomass tends towards zero. That is to say, picoplankton completely dominate warm ultra-oligotrophic systems, but not cold ones. Secondly, *D*_*s*_ can be omitted from the model when it takes a value of 1, and so the temperature-dependent model can be effectively re-written in terms of only three free parameters (i.e. $\log_{10}(C_{s})=\log_{10}(C_{s}^{m})+\log_{10}(1-e^{-\frac{C_{\text{total}}}{C_{s}^{m}}})-a_{s}e^{-b_{s}T}$).

### Model selection

The temperature-dependent model represents an extension of the temperature-independent model, and requires the estimation of (one or) two extra unknown parameters. With their additional degrees of freedom, the more complex functions are guaranteed to fit the data at least as well or better than the simpler functions, and it is important to know whether any such improvements reflect a genuine increase in the model’s ability to explain the data. A simple test of this is provided by the Akaike Information Criterion (AIC), which quantifies the balance between a model’s complexity and its ability to fit the data. The AIC can be calculated from the optimised *χ* ^2^ value, and the number of model parameters, *p*,

$$AIC=\chi^{2}+2p$$

Here *χ* ^2^ is defined as

$$\chi_{s}^{2}=\log_{10}{\left(\sigma^{2}\right)}^{-1}\sum_{i=1}^{N}(\log_{10}\left(C_{s,i}\right)-f_{s}\left(C_{\text{total}},T\right)){}^{2}$$

with *f*_*s*_(*C*_total_, *T*) representing either Eqs 1 or 2, depending on whether *T* is included as an input variable. A conservative relative error estimate of *σ* ^2^ = 10 was assigned, and *N* = 620 is the total number of observations. The AIC was calculated under the conservative assumption that the temperature-dependent function contains four (rather than three) free parameters, and was found to be smallest when fitting the temperature-dependent function to both *C*_pico_ and *C*_nano,pico_ (Table 3). This indicates that the temperature-dependent function has more explanatory power, and that it should be preferred [32].

A more practical test of a model’s skill is to establish if any improvements in fit are also matched by increased predictive skill in relation to independent data. This was done in a cross-validation experiment, in which observations were divided into training data and test data. To ensure the independence of the test data from the training data, the observations were categorised into 24 subsets according to the research programme and the prevailing oceanographic conditions (regional or seasonal—see Table 1). The regressions were then repeated 24 times, each time omitting one of the 24 observation subsets. In each case the fitted functions were used to predict the size-fractionated biomasses in the omitted test datasets on the basis of observed chlorophyll *a* and, optionally, temperature. The combined sum-of-squared-errors for all iterations provides a quantitative estimate of the predictive skill for each model (Table 3).

**Fig 3:**
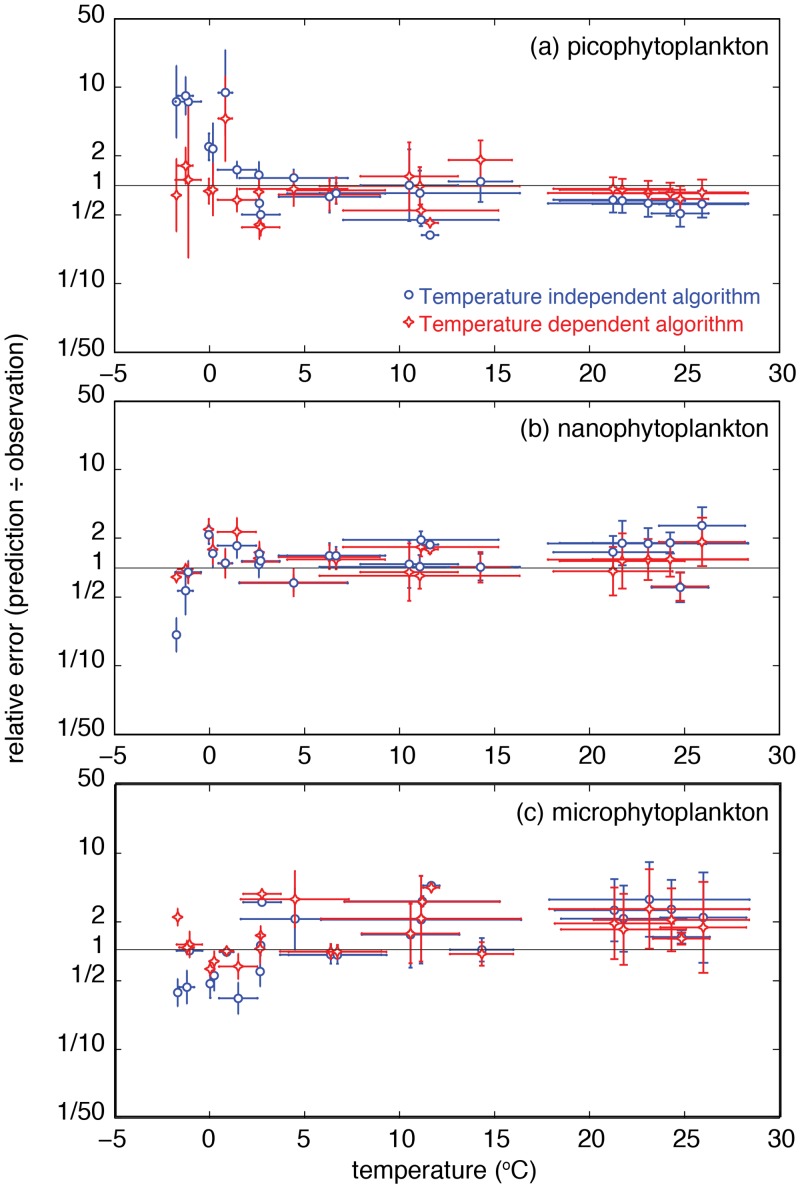
Relative error of the temperature-dependent and temperature-independent functions when used to predict size-class biomasses for independent, unassimilated observations. Dots represent the mean temperature and geometric mean relative error for each data subset, with error bars showing ±1 standard deviation for temperature, and ${}_{÷}^{\times }$1 geometric standard deviation for the relative error.

The results from this experiment are shown in Fig 3. At temperatures below ∼2°C the temperature-independent model dramatically overestimates picoplankton biomass, while underestimating nanoplankton and microplankton biomass. Switching to the temperature-dependent model allows a marked improvement in the ability to predict the community structure at very cold temperatures. Overall, the temperature-dependent model has 39% (pico-), 27% (nano-) and 19% (micro-) better predictive skill in terms of the sum-of-squared-errors for the independent data, relative to the temperature-independent model.

### Global patterns

Noting that the temperature-dependent model was consistently better in terms of fit to the data and predictive skill, the two models were used to estimate annual mean global phytoplankton community structure from climatological satellite estimates of surface chlorophyll *a* (SeaWiFS, e.g. [8, 10]) and sea-surface temperature (SST) (NOAA Optimum Interpolation SST V2 [33]), for the years 1998 to 2003. (Given the non-linearities of Eqs 1 and 2, results based on these composite inputs should be considered as illustrative only—a more quantitative analysis should be based on daily input data.)

**Fig 4:**
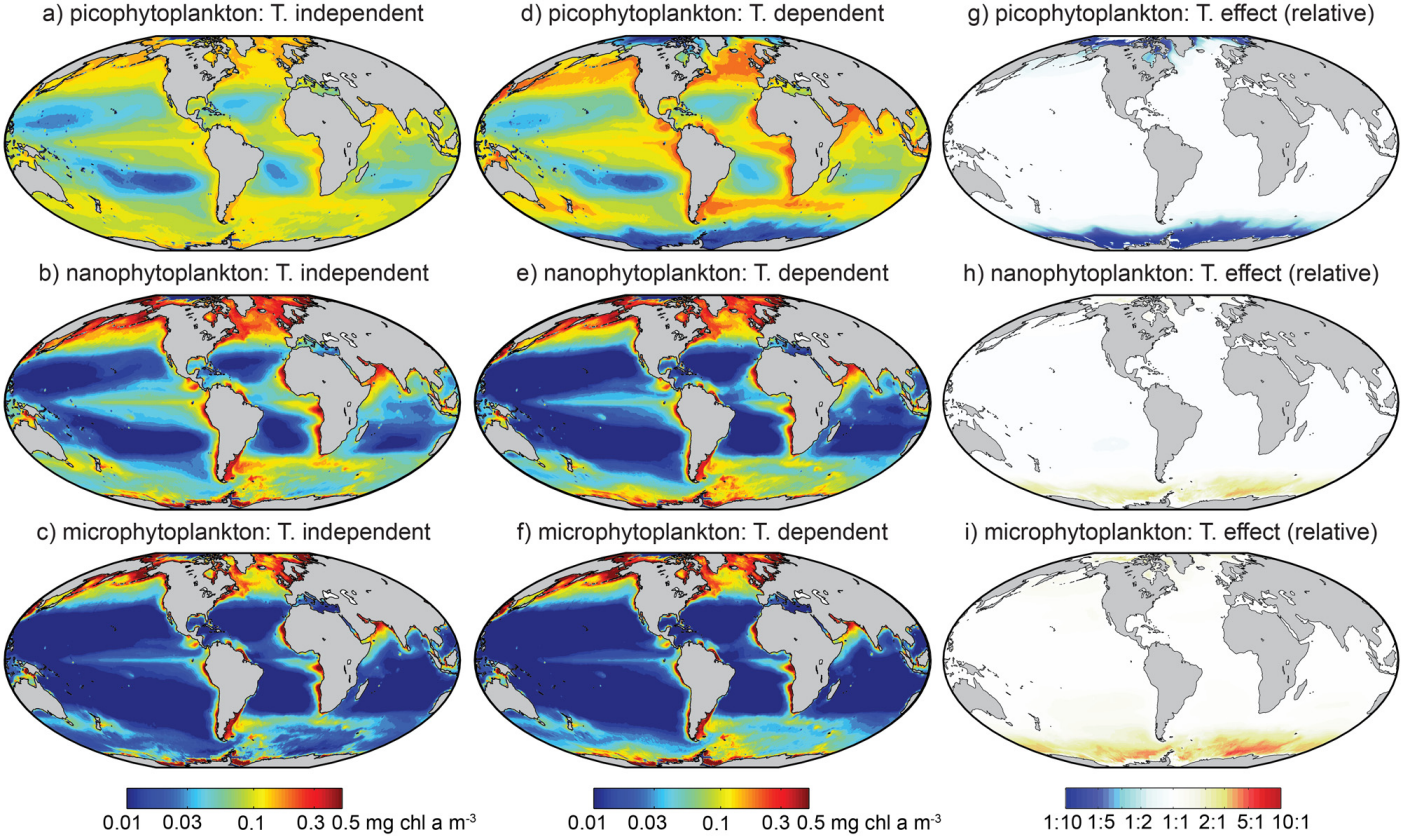
Satellite-derived estimates of annual mean surface chlorophyll *a* biomass in three phytoplankton size classes. Panels (a-c): Temperature-independent functions (Eq 1). Panels (d-f): Temperature-dependent functions (Eq 2). Panels (g-i): Relative change in biomass attributable to temperature, calculated by removing the two chlorophyll-dependent terms from Eq 2.

**Fig 5:**
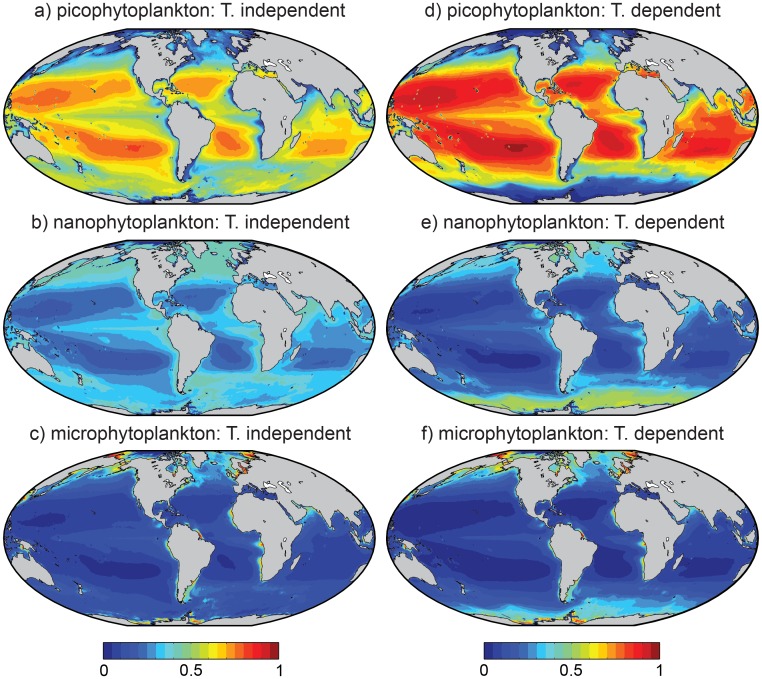
Satellite-derived estimates of the fractional contribution of the three phytoplankton size classes to annual mean total surface chlorophyll *a* biomass. Panels (a-c): Temperature-independent functions (Eq 1). Panels (d-f): Temperature-dependent functions (Eq 2).

Global maps of the surface biomass in each size fraction are shown for the temperature-independent model in Fig 4a–4c, and for the temperature-dependent model in Fig 4d–4f. The third column shows only the temperature-dependent component of Eq 2, which gives the relative effect of that term on the biomass in each size fraction. Fig 5 shows the estimated contribution of each size class to the total surface chlorophyll *a*, for the temperature-independent (panels a-c) and temperature-dependent (panels d-f) models.

Direct temperature (or other correlated) effects lead to estimates of Southern Ocean and Arctic picoplankton biomass that are as much as an order of magnitude lower relative to higher-latitude regions with equivalent total biomass (Fig 4g). Microplankton and nanoplankton biomass each show a modest compensatory increase in the same regions (Fig 4h and 4i). The lack of any direct temperature dependence at low latitudes indicates that any differences between the two functions at low latitudes are attributable to differences in the temperature-independent parameters $C_{s}^{m}$ and *D*_*s*_ (Table 3). The temperature-dependent function, for example, correctly predicted slightly higher picoplankton chlorophyll concentrations at low latitudes (Fig 3a), because it was not so strongly constrained by the low picoplankton chlorophyll concentrations at high latitudes. This conclusion is supported by the fact that the higher values at low latitudes were also returned by the temperature-independent function when all observations taken at temperatures below ∼2°C were excluded from the analysis (result not shown).

In terms of the relative contribution of each size class to total biomass (Fig 5), the temperature-dependent model suggests that the picoplankton contribute a much greater fraction of total chlorophyll *a* at low latitudes (i.e. ≲ 40° N or S) than is indicated by the temperature-independent model. At the same time, the exclusion of picoplankton at very cold temperatures increases the relative importance of the nanoplankton and microplankton classes at higher latitudes. Given that the estimates shown in Fig 5 are based on annual mean total surface chlorophyll *a*, the relative contribution of different groups is likely to change throughout the year.

**Fig 6:**
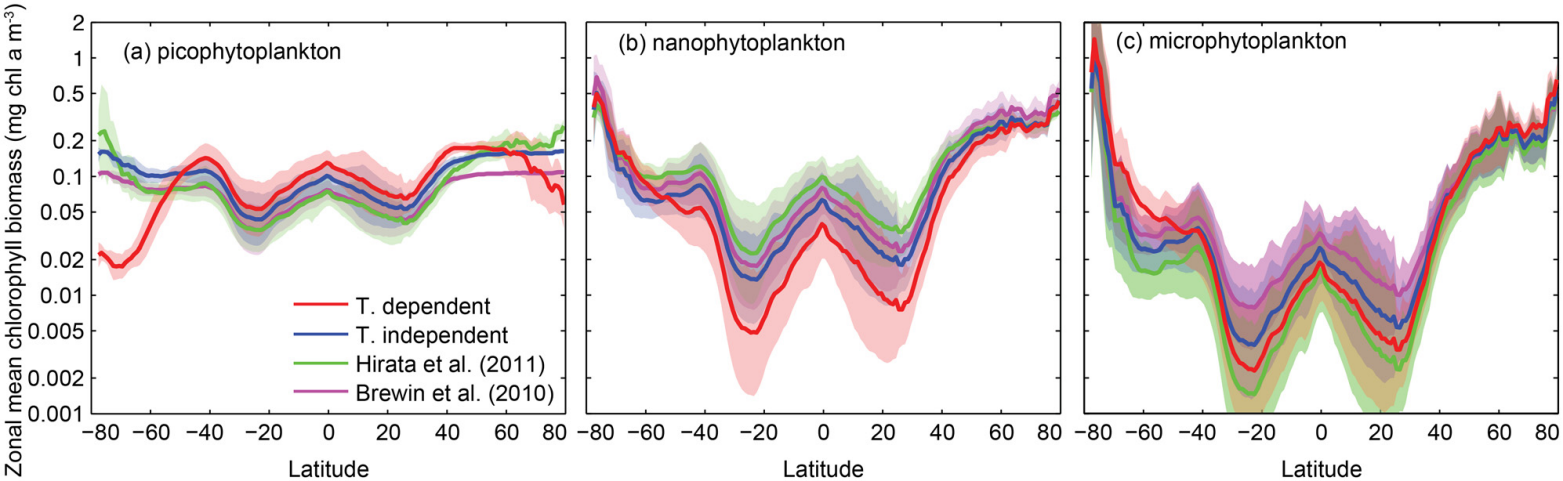
Four satellite-derived estimates for the zonal geometric mean surface chlorophyll *a* biomass (${}_{÷}^{\times }$1 geometric standard deviation) in (a) picoplankton, (b) nanoplankton, and (c) microplankton size classes.

Satellite-derived estimates of zonal geometric mean biomasses (${}_{÷}^{\times }$1 geometric standard deviation) are shown for the temperature-independent and temperature-dependent functions in Fig 6. For the purposes of comparison, zonal geometric means and standard deviations from two other, temperature-independent functions are also shown [9, 10], using the parameter values reported in those studies.

The four functions follow generally similar trends. Picoplankton are the least variable with latitude, with a range of ∼1 order of magnitude. Variability is progressively greater in the larger size classes, with nanoplankton and microplankton varying by ∼2 and ∼3 orders of magnitude, respectively. There are however distinct qualitative differences in the picoplankton estimates from the temperature-dependent and temperature-independent functions. The temperature-dependent function is unique in showing a general decline in picoplankton biomass polewards of 40° N or S, contrary to the overall trend in total biomass. The temperature-dependent estimates are thus markedly lower than all the temperature-independent estimates in the Southern and Arctic Oceans.

## Discussion

The analysis presented above conforms with the view that the size-structure of marine phytoplankton communities is very strongly dependent on total community biomass, primarily as a function of nutrient supply and primary production [4, 17]. Nonetheless, there is also a significant additional effect that becomes apparent at cold temperatures. In cold and dark high-latitude environments, small cells become disproportionately rare relative to the total community biomass. While it should be noted that the dataset used in this study includes only a limited number of observations representative of these extreme environments, the observed trends are supported by a number of independent studies from high latitudes that have shown a decline in picoplankton biomass at low temperatures [12–15]. Nonetheless, of the 76 observations taken in waters below 2°C, 58 come from the same time-series location off the west coast of the Antarctic Peninsula [18], and further observations will therefore be required to increase confidence in the proposed trends over the broader polar regions.

It is also important to note that while temperature was found to be an important predictive variable, this does not necessarily mean that the observed changes in phytoplankton community structure were directly attributable to temperature, *per se*. Cold temperatures are well correlated with a range of other environmental factors that have a detrimental effect on phytoplankton growth, including weak incident solar radiation and deep mixing, and the observed effects may be attributable to any one or many such factors. This uncertainty is not helped by the fact that the temperature-dependent term added in Eq 2 is simply an *ad hoc* extension that provides no mechanistic insight into the cause of the observed trends. Nonetheless, the statistical analysis presented here indicates that algorithms designed to provide global scale estimates of phytoplankton community structure from satellite estimates of total chlorophyll biomass [8, 9] may show significant improvement at high latitudes if remotely-sensed SST data are included as an input variable. Again, the lack of an equivocal causative role for temperature suggests that the observed trends might also be captured through other approaches based on alternative input variables, such as directly observed light absorption and backscattering properties [34–36]. This possibility was not investigated here.

The observed anomalies at high latitudes also have implications for the estimation of total chlorophyll *a* concentrations from satellite data. Ocean colour algorithms typically operate on the basis of an assumed relationship between total chlorophyll *a* concentrations and the ratio of blue-to-green reflected light (the remote sensing reflectance, *R*_*rs*_). This ratio is driven by the absorption and backscattering of different wavelengths of light, which both vary as a function of cell size [16]. Microplankton, for example, may absorb up to eight times less light per molecule of chlorophyll *a* than picoplankton [37]. Changes in community structure can therefore cause *R*_*rs*_ to vary independently of total chlorophyll *a*. The disproportionately low abundances of picoplankton in the Southern Ocean may therefore help to explain the tendency of some satellite algorithms to yield unrealistically low chlorophyll *a* estimates in this region [38]. In future a better representation of biomass-independent changes in phytoplankton community structure may help to reduce these discrepancies.

At present there remains little evidence of a large temperature dependence at latitudes below ∼40° N or S, where community structure appears to be very well determined by nutrient supply alone [4, 9]. Temperature effects in a warming world may therefore be restricted to the biogeographical spread of smaller size classes to higher latitudes [15]. Such changes may nonetheless have important implications with regard to the biogeochemical function of marine ecosystems in these areas, as the observed exponential decline of picophytoplankton at cold temperatures reflects a similar decrease in the efficiency of nutrient recycling [39]. An extension of picoplankton habitats to higher latitudes with warmer temperatures and stronger stratification could potentially lead to strengthening of the microbial loop and diminished export efficiency in regions previously characterised by larger phytoplankton and the efficient vertical export of biomass. Similarly, the encroachment of picoplankton into higher latitudes currently characterised by short, direct food chains based on fast-growing nanoplankton [2] could potentially lead to diminished trophic-transfer to larger size classes and a decline in productivity at higher trophic levels.

## References

[1] Smetacek VS. Role of sinking in diatom life-history cycles: ecological, evolutionary and geological significance. Marine Biology. 1985;84:239–251. 10.1007/BF00392493

[2] Cushing DH. A difference in structure between ecosystems in strongly stratified waters and in those that are only weakly stratified. Journal of Plankton Research. 1989;11(1):1–13. 10.1093/plankt/11.1.1

[3] Edwards KF, Thomas MK, Klausmeier CA, Litchman E. Allometric scaling and taxonomic variation in nutrient utilization traits and maximum growth rate of phytoplankton. Limnology and Oceanography. 2012;57(2):554–566. 10.4319/lo.2012.57.2.0554

[4] Marañón E, Cermeño P, Latasa M, Tadonléké RD. Temperature, resources, and phytoplankton size structure in the ocean. Limnology and Oceanography. 2012;5:1266–1278.

[5] Litchman E, Klausmeier CA, Yoshiyama K. Contrasting size evolution in marine and freshwater diatoms. Proceedings of the National Academy of Sciences of the United States of America. 2009;106:2665–2670. 10.1073/pnas.0810891106 19202058 PMC2650323

[6] Thingstad TF, Sakshaug E. Control of phytoplankton growth in nutrient recycling ecosystems. Theory and terminology. Marine Ecology Progress Series. 1990;63:261–272. 10.3354/meps063261

[7] Armstrong RA. Grazing limitation and nutrient limitation in marine ecosystems: Steady state solutions of an ecosystem model with multiple food chains. Limnology and Oceanography. 1994;39:597–608. 10.4319/lo.1994.39.3.0597

[8] Uitz J, Claustre H, Morel A, Hooker SB. Vertical distribution of phytoplankton communities in open ocean: An assessment based on surface chlorophyll. Journal of Geophysical Research. 2006;111(C08005).

[9] Brewin RJW, Sathyendranath S, Hirata T, Lavender SJ, Barciela RM, Hardman-Mountford NJ. A three-component model of phytoplankton size class for the Atlantic Ocean. Ecological Modelling. 2010;221:1472–1483. 10.1016/j.ecolmodel.2010.02.014

[10] Hirata T, Hardman-Mountford NJ, Brewin RJW, Aiken J, Barlow R, Suzuki K, et al Synoptic relationships between surface chlorophyll-*a* and diagnostic pigments specific to phytoplankton functional types. Biogeosciences. 2011;8:311–327. 10.5194/bg-8-311-2011

[11] Lopez-Urrutia A, Moran XAG. Temperature affects the size-structure of phytoplankton communities in the ocean. Limnology and Oceanography. 2015;.

[12] Sosik HM, Olson RJ. Phytoplankton and iron limitation of photosynthetic efficiency in the Southern Ocean during late summer. Deep-Sea Research I. 2002;49:1195–1216. 10.1016/S0967-0637(02)00015-8

[13] Li WKW, McLaughlin FA, Lovejoy C, Carmack EC. Smallest algae thrive as the Arctic Ocean freshens. Science. 2009;326:539 10.1126/science.1179798 19900890

[14] Morán XAG, López-Urrutia Á, Calvo-Díaz A, Li WKW. Increasing importance of small phytoplankton in a warmer ocean. Global Change Biology. 2010;16:1137–1144. 10.1111/j.1365-2486.2009.01960.x

[15] Flombaum P, Gallegos JL, Gordillo RA, Rincón J, Zabala LL, Jiao N, et al. Present and future global distributions of the marine cyanobacteria *Prochlorococcus* and *Synechococcus*. Proceedings of the National Academy of Sciences of the United States of America. 2013;. 10.1073/pnas.1307701110 PMC3683724 23703908

[16] Sathyendranath S, Cota G, Stuart V, Maass H, Platt T. Remote sensing of phytoplankton pigments: a comparison of empirical and theoretical approaches. International Journal of Remote Sensing. 2001;22(2):249–273. 10.1080/014311601449925

[17] Marañón E, Cermeño P, Latasa M, Tadonléké RD. Resource supply alone explains the variability of marine phytoplankton size structure. Limnology and Oceanography. 2015;.

[18] Clarke A, Meredith MP, Wallace MI, Brandon MA, Thomas DN. Seasonal and interannual variability in temperature, chlorophyll and macronutrients in northern Marguerite Bay, Antarctica. Deep-Sea Research II. 2008;55:1988–2006. 10.1016/j.dsr2.2008.04.035

[19] Froneman PW, Pakhomov A, Balarin MG. Size-fractionated phytoplankton biomass, production and biogenic carbon flux in the eastern Atlantic sector of the Southern Ocean in late austral summer 1997–1998. Deep-Sea Research II. 2004;51:2715–2729. 10.1016/j.dsr2.2002.09.001

[20] Froneman PW, Laubscher RK, McQuaid CD. Size-fractionated primary production in the south Atlantic and Atlantic sectors of the Southern Ocean. Journal of Plankton Research. 2001;23:611–622. 10.1093/plankt/23.6.611

[21] Faila M, Semeneh M, Oriol L. Size-fractionated phytoplankton biomass and species composition in the Indian sector of the Southern Ocean during austral summer. Journal of Marine Systems. 1998;17:179–194. 10.1016/S0924-7963(98)00037-2

[22] Boyd PW, Watson AJ, Law CS, Abraham ER, Trull T, Murdoch R, et al A mesoscale phytoplankton bloom in the polar Southern Ocean stimulated by iron fertilization. Nature. 2000;407:695–702. 10.1038/35037500 11048709

[23] Imai K, Nojiri Y, Tsurushima N, Saino T. Time series of seasonal variation of primary productivity at station KNOT (44°N, 155°E) in the sub-arctic western North Pacific. Deep-Sea Research II. 2002;49:5395–5408. 10.1016/S0967-0645(02)00198-4

[24] Tsuda A, Takeda S, Saito H, Nishioka J, Nojiri Y, Kudo I, et al A mesoscale iron enrichment in the western subarctic Pacific induces a large centric diatom bloom. Science. 2003;300:958–961. 10.1126/science.1082000 12738858

[25] Marañón E, Holligan PM, Barciela R, González N, Mouriño B, Pazó MJ, et al Patterns of phytoplankton size structure and productivity in contrasting open-ocean environments. Marine Ecology Progress Series. 2001;216:43–56. 10.3354/meps216043

[26] Cermeño P, Marañón E, Pérez V, Serret P, Fernández E, Castro CG. Phytoplankton size structure and primary production in a highly dynamic coastal ecosystem (Ría de Vigo, NW-Spain): Seasonal and short-time scale variability. Estuarine, Coastal and Shelf Science. 2006;67:251–266. 10.1016/j.ecss.2005.11.027

[27] Lara-Lara JR, Banzán-Guzmán C. Distribution of chlorophyll and primary production by size classes along the Mexican Pacific coast. Ciencias Marinas. 2005;31:11–21.

[28] Latasa M, Bidigare RM. A comparison of phytoplankton populations of the Arabian Sea during the Spring Intermonsoon and Southwest Monsoon of 1995 as described by HPLC-analyzed pigments. Deep-Sea Research II. 1998;45:2133–2170. 10.1016/S0967-0645(98)00066-6

[29] Devred E, Sathyendranath S, Stuart V, Maass H, Ulloa O, Platt T. A two-component model of phytoplankton absorption in the open ocean: Theory and applications. Journal of Geophysical Research. 2006;111(C03011).

[30] Brewin RJW, Sathyendranath S, Tilstone G, Lange PK, Platt T. A multicomponent model of phytoplankton size structure. Journal of Geophysical Research: Oceans. 2014;119.

[31] Marañón E. Cell Size as a Key Determinant of Phytoplankton Metabolism and Community Structure. Annual Review of Marine Science. 2015;7 25062405 10.1146/annurev-marine-010814-015955

[32] Johnson JB, Omland KS. Model selection in ecology and evolution. Trends in Ecology and Evolution. 2004;19(2):101–108. 10.1016/j.tree.2003.10.013 16701236

[33] Reynolds RW, Rayner NA, Smith TM, Stokes DC, Wang W. An improved in situ and satellite SST analysis for climate. Journal of Climate. 2002;15:1609–1625. 10.1175/1520-0442(2002)015<1609:AIISAS>2.0.CO;2

[34] Kostadinov TS, Siegel DA, Maritorena S. Retrieval of the particle size distribution from satellite ocean color observations. Journal of Geophysical Research. 2009;114:C09015 10.1029/2009JC005303

[35] Mouw CB, Yoder JA. Optical determination of phytoplankton size composition from global SeaWiFS imagery. Journal of Geophysical Research. 2010;115 10.1029/2010JC006337

[36] Roy S, Sathyendranath S, Bouman H, Platt T. The global distribution of phytoplankton size spectrum and size classes from their light-absorption spectra derived from satellite data. Remote Sensing of Environment. 2013;139:185–197. 10.1016/j.rse.2013.08.004

[37] Dierssen HM. Perspectives on empirical approaches for ocean color remote sensing of chlorophyll in a changing climate. Proceedings of the National Academy of Sciences of the United States of America. 2010;107(40):17073–17078. 10.1073/pnas.0913800107 20861445 PMC2951429

[38] Dierssen HM, Smith RC. Bio-optical properties and remote sensing ocean color algorithms for Antarctic Peninsula waters. Journal of Geophysical Research. 2000;105(C11):26,301–26,312. 10.1029/1999JC000296

[39] Henson SA, Sanders R, Madsen E, Morris PJ, Le Moigne F, Quartly GD. A reduced estimate of the strength of the ocean’s biological carbon pump. Geophysical Research Letters. 2011;38 10.1029/2011GL046735

